# Preclinical pharmacokinetic characterization of (R)-ketamine injection, a novel antidepressant glutamatergic agent

**DOI:** 10.3389/fphar.2025.1699304

**Published:** 2025-11-21

**Authors:** Rui Wang, Yongchao Jin, Bingjie Zou, Li Ding

**Affiliations:** 1 Department of Pharmaceutical Analysis, China Pharmaceutical University, Nanjing, China; 2 Jiangsu Nhwa Pharmaceutical Co., Ltd., Xuzhou, China; 3 Nanjing Clinical Technology Co., Ltd., Nanjing, China

**Keywords:** (R)-ketamine, preclinical pharmacokinetics, distribution, metabolism, excretion

## Abstract

**Introduction:**

Ketamine is a significant class of antidepressant drugs, and the pharmacokinetic and pharmacodynamic characteristics of its enantiomers exhibit differences. Among them, intravenous infusion of (R)-ketamine can effectively alleviate depressive symptoms in patients, offering the advantages of prolonged duration of action and minimal side effects. The extensive preclinical pharmacokinetic (PK) studies of (R)-ketamine were conducted to support its further development.

**Methods:**

This study investigated the preclinical pharmacokinetic behavior of (R)-ketamine across different dose groups through single- and multiple-dose pharmacokinetic studies in rats and dogs, along with tissue distribution studies in Sprague-Dawley (SD) rats.*In vitro* metabolism study using microsomes from mice, rats, dogs, monkeys, and humans were conducted to evaluate metabolic stability and obtain metabolite profiles. The excretion studies in rats (7.5 mg/kg dose group) were performed to elucidate the primary elimination pathways of (R)-ketamine. The allometric scaling approach was employed to predict human plasma total clearance based on preclinical data.

**Results:**

(R)-ketamine exhibits nonlinear pharmacokinetics in SD rats and Beagle dogs, with plasma exposure increasing disproportionately to dose after both single and multiple intravenous administrations. The highest tissue exposure to (R)-Ketamine was found in the fat and kidney of the rats. The (R)-ketamine concentration ratio of brain/plasma was 2.25 for the rats, indicating effective blood-brain barrier penetration and significant brain distribution of (R)-ketamine. (R)-ketamine undergoes rapid metabolism in liver microsomes from mice, rats, dogs, monkeys and humans. The predominant metabolite identified in human, monkey and mice liver microsomes was (R)-norketamine and demethylated and mono-oxidized metabolites in rats and dogs. (R)-ketamine is primarily excreted via bile, accounting for 3.613% of the dose within 72 h, significantly higher than in feces (0.327%) or urine (0.030%). The main elimination pathway may be metabolic elimination, and eventually excreted in the form of metabolites. The predicted human plasma total clearance of 29.93 mL/min/kg is close to reported value.

**Conclusion:**

The preclinical pharmacokinetic characteristics of (R)-ketamine injection have been thoroughly investigated, and these findings can provide valuable information for predicting the first-in-human dose and designing Phase I clinical trials for (R)-ketamine injection.

## 1 Introduction

Depression is a mental disorder characterized primarily by prolonged periods of low mood, often accompanied by delusions and hallucinations. In severe cases, patients may exhibit self-harm or suicidal behaviors. According to statistics, approximately 3.8% of the global population suffers from depression (WHO, 2023). In recent years, a variety of pharmacological treatments have emerged to address this condition. Depression is associated with dysregulation of glutamate function in specific brain regions (Abdallah et al., 2018; Krystal et al., 2019). Glutamate, a common excitatory neurotransmitter, exhibits abnormally elevated levels in depression, which reduces synaptic connectivity and leads to deficits in γ-aminobutyric acid (GABA) function (Sanacora et al., 2012; Marwaha et al., 2023). Among the most common classes of drugs used to treat depression are N-methyl-D-aspartate (NMDA) receptor modulators, which primarily exert their therapeutic effects by regulating the GABA pathway (Lin et al., 2021). Ketamine, a glutamatergic agent, acts as an antagonist of NMDA receptors in the brain and shows promising potential in the treatment of depression (Autry et al., 2011; Reese and Kavalali, 2015). Compared to other NMDA receptor modulators, such as dextromethorphan and methadone, ketamine demonstrates superior affinity when binding to NMDA receptors (Taylor et al., 2016; Fava et al., 2022; Autry et al., 2011; Yang et al., 2020).

The distinct pharmacokinetic and pharmacodynamic profiles of ketamine enantiomers have prompted significant research into their differential antidepressant effects (Yang et al., 2019; Zanos et al., 2023a; Mion and Villevieille, 2013; Johnston et al., 2023; Zanos et al., 2023b). In terms of rapid antidepressant action, preclinical studies have demonstrated that both enantiomers exhibit rapid antidepressant-like effects (Yokoyama et al., 2020; Singh et al., 2016). However, behavioral studies indicate that (R)-ketamine possesses stronger anti-anhedonic and anti-apathetic effects compared to (S)-ketamine, as well as rapid behavioral effects when used in combination therapies (Anna and Paucha-Poniewiera, 2022; Zhang et al., 2014). Furthermore, extensive preclinical research has shown that (R)-ketamine is more effective than (S)-ketamine in reducing depressive behaviors (Chang et al., 2019). Regarding long-term antidepressant effects, (R)-ketamine exhibits a more sustained impact (Zhang et al., 2014; Murrough et al., 2013). Specifically, the anti-anhedonic and anti-apathetic effects of (R)-ketamine persist for up to 7 days post-administration, whereas those of (S)-ketamine last for only 3 days (Anna et al., 2022). In terms of side effects, preclinical studies suggest that (S)-ketamine is associated with a higher incidence of adverse reactions compared to the racemic mixture or (R)-ketamine, presumably due to its higher NMDAR (N-methyl-D-aspartate receptor) inhibitory potency (Acevedo-Diaz et al., 2020; Maggie et al., 2019; Popova et al., 2019).

Other studies have demonstrated that ketamine treatment effectively alleviates depressive symptoms in patients, including those with treatment-resistant depression (Gould and Thompson, 2019). However, in most patients who respond well to a single dose of ketamine, the therapeutic effects typically diminish within 2 weeks. Researchers have explored various strategies to prolong these effects, including the administration of riluzole, lithium, and repeated doses of ketamine. Among these, repeated ketamine dosing has proven to be the most effective method, suggesting that maintenance therapy with ketamine may be necessary (Krystal et al., 2023). Currently, common administration routes include intravenous injection, intramuscular injection, oral tablets, and intranasal delivery of esketamine. Notably, intravenous infusion of ketamine has been shown to significantly alleviate depressive symptoms, offering the advantages of prolonged efficacy and fewer side effects. Therefore, the development of (R)-ketamine injectable formulations is of considerable importance. The majority of published pharmacokinetic and pharmacodynamic studies on ketamine have been conducted with racemic ketamine. Recent animal studies have revealed that (R)-ketamine exhibits distinct mechanisms of action and superior safety profiles in the antidepressant domain compared to (S)-ketamine. However, pharmacokinetic investigations in this area remain limited. So, Jiangsu Nhwa Pharmaceutical Co., Ltd. conducted the preclinical research on (R)-ketamine injection to investigate its preclinical pharmacokinetic characterization.

## 2 Materials and methods

### 2.1 Chemicals, reagents, and materials

(R)-ketamine hydrochloride (99.2% purity) and (R)-2-amino-2-(2-chlorophenyl) cyclohexanone (99.7% purity) were provided by Jiangsu Enhua Pharmaceutical Co., Ltd. The internal standard, tolbutamide, was supplied by Sigma-Aldrich (Shanghai) Trading Co., Ltd. Preclinical animals were purchased from standard suppliers in China and were accompanied by certificates of production and use for experimental animals. HPLC-grade acetonitrile, methanol, isopropanol, and dimethyl sulfoxide (DMSO) were purchased from Sigma-Aldrich (St. Louis, MO, United States). Sodium chloride injection (0.9%) was provided by Sichuan Kelun Pharmaceutical Co., Ltd. KH_2_PO_4_, K_2_HPO_4_·3H_2_O, MgCl_2_·6H_2_O, and concentrated hydrochloric acid were supplied by Sinopharm Chemical Reagent Co., Ltd. Warfarin, phenacetin, and verapamil were commercially available standards. Plasma containing ethylenediaminetetraacetic acid dipotassium salt (EDTA-K_2_) as anticoagulant from SD rats, beagle dogs, and cynomolgus monkeys was provided by Innostar Bio-technology (Nantong) Co., Ltd. Plasma containing EDTA-K_2_ as anticoagulant from mice was supplied by Fuhe (Shanghai) Biotechnology Co., Ltd. Human plasma was obtained from the People’s Hospital of Weifang High-Tech Industrial Development Zone. Blank urine and feces from SD rats were provided by Innostar Bio-technology (Nantong) Co., Ltd., while blank bile from SD rats was supplied by Fuhe (Shanghai) Biotechnology Co., Ltd.Liver microsomes from humans, dogs, rats, and mice were purchased from Xeno Tech, and cynomolgus monkey liver microsomes were obtained from RILD (Reid Liver Disease Research (Shanghai) Co., Ltd.). Deionized water was prepared using a Millipore Gradient Water Purification System (Bedford, MA, United States). All other chemicals and reagents were of research grade and used without further purification.

### 2.2 Animals

SD rats were purchased from Zhejiang Vital River Laboratory Animal Technology Co., Ltd. During the experimental period, the animals were housed in an environment with a temperature range of 20 °C–26 °C and a relative humidity of 40%–70%, meeting the required standards for housing conditions. (Permit No. SYXK 2018–0034).

Beagle dogs were provided by Beijing Marshall Biotechnology Co., Ltd. Throughout the study, the animals were maintained in an environment with a temperature range of 18 °C–26 °C and a relative humidity of 40%–70%, ensuring compliance with the required housing conditions. (Permit No. SYXK 2018–0033).

Jiangsu Nhwa Pharmaceutical Co., Ltd. commissioned InnoStar Bio-tech Nantong Co., Ltd. to conduct preclinical animal studies involving animal ethics in this research, the corresponding declaration and official statements can be found in the Supplementary Material. So, the animal experiments in this study were conducted at Innostar Bio-technology (Nantong) Co., Ltd., in facilities accredited by the International Council for Laboratory Animal Science (ICLAS) and approved by the Institutional Animal Care and Use Committee (IACUC). The corresponding IACUC approval numbers are as follows: H21025PK3: 2021-714a, H21025PK4: 2021–711, H21025TD1: 2021-715a and H21025EX1: 2021–712.

### 2.3 Drug preparation before administration

An amount of (R)-ketamine was dissolved in 0.9% sodium chloride injection solution. Hydrochloric acid was added to adjust the pH of the intermediate drug solution (pH 5) for intravenous administration in rats and dogs. This prepared solution was used for pharmacokinetic studies, tissue distribution studies, and *in vivo* excretion studies.

### 2.4 Quantitative sample analysis

An aliquot of 20 μL of plasma or tissue homogenat or fecal homogenates samples was precipitated with 160 μL of acetonitrile containing 200 ng/mL internal standard (IS, tolbutamide) and centrifuged at 4700 *g* at 4 °C for 10 min after vortexing for 10 min. The clear supernatant (100 μL) was mixed with 100 μL of distilled water before injection into the LC-MS/MS system for analysis. Chromatographic separation was achieved on an ACQUITY UPLC HSS T3 column (50 mm × 2.1 mm, 1.8 μm) using an LC-ACQUITY^TM^ Ultra Performance system (Waters, USA). The mobile phase consisted of (A) water containing 2 mM ammonium acetate and (B) acetonitrile containing 2 mM ammonium acetate. The column temperature was maintained at 40 °C with a flow rate of 0.6 mL/min. The gradient program started at 20% B, increased to 95% B over 1.2 min, held for 0.9 min, then returned to 20% B for 0.4 min column re-equilibration. Mass spectrometric detection was performed using a QTRAP 6500 system (AB SCIEX) with electrospray ionization in positive mode (ESI+). Multiple reaction monitoring (MRM) transitions were m/z 237.9→163.0 for (R)-ketamine and m/z 271.1→154.8 for the internal standard tolbutamide. The quantitation range was 1–2000 ng/mL for both rat and dog studies.

An aliquot of 50 μL of urine or bile samples was extracted with 950 μL of methyl tertiary-butyl ether and centrifuged at 4700 *g* at 4 °C for 10 min after vortexing for 10 min. The clear supernatant (100 μL) was evaporated to dryness under nitrogen stream for 15 min, followed by reconstitution with 250 μL of 50% acetonitrile containing 10 ng/mL IS (tolbutamide) before injection into the LC-MS/MS system for analysis. Chromatographic separation was achieved on an XBridge BEH C18 column (50 mm × 2.1 mm, 2.1 μm) using an LC-ACQUITYTM Ultra Performance system (Waters, USA). The mobile phase consisted of (A) water containing 0.2% formic acid and 5 mM ammonium formate and (B) acetonitrile containing 0.1% formic acid. The column temperature was 40 °C with a flow rate of 0.35 mL/min. The gradient program started at 5% B, increased to 40% B over 2 min, then to 95% B in 2.2 min, held for 0.8 min, followed by re-equilibration at 5% B for 0.1 min. MS detection used the same instrument and ionization conditions, with identical MRM transitions. The quantitation range was 1–2000 ng/mL.

### 2.5 Method validation

The LC-MS/MS methods for the determination of (R)-ketamine in biological samples was validated in accordance with the U.S. Food and Drug Administration (FDA) Guidance for Bioanalytical Method Validation (2018). The validation included assessments of selectivity, carryover, matrix effects, recovery, linearity, lower limit of quantification (LLOQ), upper limit of quantification (ULOQ), accuracy, precision, and stability.

### 2.6 Pharmacokinetic studies in rats and dogs

The pharmacokinetic characteristics of (R)-ketamine hydrochloride injection in SD rats were investigated following intravenous administration via the tail vein. Groups 1 to 3 were administered intravenous injections of (R)-ketamine hydrochloride at doses of 2.5, 7.5, and 25 mg/kg, respectively. Groups 1 and 3 received a single dose, while Group 2 receiving multiple dosing, with once-daily dosing for seven consecutive days. Eighteen rats were randomly divided into three groups, with six rats (male: female = 1:1) in each group. Blood samples were collected at the following time points: pre-dose (0), 0.033 h (2 min), 0.167 h (10 min), 0.5 h (30 min), 1 h, 1.5 h, 2 h, 3 h, 4 h, 5 h, 6 h, and 8 h post single dose; pre-dose (0),0.033 h (2 min), 0.167 h (10 min), 0.5 h (30 min), 1 h, 1.5 h, 2 h, 3 h, 4 h, 5 h, 6 h, 8 h, pre-second dose (24 h), pre-third dose (48 h), pre-fourth dose (72 h), pre-fifth dose (96 h), pre-sixth dose (120 h), pre-seventh dose (144 h) post multiple dose and at 0.033 h (2 min), 0.167 h (10 min), 0.5 h (30 min), 1 h, 1.5 h, 2 h, 3 h, 4 h, 5 h, 6 h, and 8 h post final dose.Approximately 250 μL of whole blood was collected through the jugular vein of rats and transferred to the tube containing EDTA-K_2_.The gathered blood samples were kept on ice until centrifugation. Plasma samples were harvested by centrifugation at 2000 *g* for 10 min at 4 °C.

The pharmacokinetic characteristics of (R)-ketamine hydrochloride injection in Beagle dogs were investigated following intravenous administration via peripheral veins. Groups 1 to 3 were administered intravenous injections of (R)-ketamine hydrochloride at doses of 1, 3, and 10 mg/kg, respectively. Groups 1 and 3 received a single dose, while Group 2 receiving multiple dosing, with once-daily dosing for seven consecutive days. Blood samples were collected at the following time points: pre-dose (0), 0.0833 h (5 min), 0.167 h (10 min), 0.5 h (30 min), 1 h, 1.5 h, 2 h, 3 h, 4 h, 6 h, 8 h, and 12 h post single dose; pre-dose (0), 0.0833 h (5 min), 0.167 h (10 min), 0.5 h (30 min), 1 h, 1.5 h, 2 h, 3 h, 4 h, 6 h, 8 h, 12 h, pre-second dose (24 h), pre-third dose (48 h), pre-fourth dose (72 h), pre-fifth dose (96 h), pre-sixth dose (120 h), pre-seventh dose (D7) post multiple dose and at 0.0833 h (5 min), 0.167 h (10 min), 0.5 h (30 min), 1 h, 1.5 h, 2 h, 3 h, 4 h, 6 h, 8 h, and 12 h post final dose.Approximately 1.50 mL of whole blood was collected through the peripheral venous access of dogs and transferred to the tube containing EDTA-K_2_.The gathered blood samples were kept on ice until centrifugation. Plasma samples were harvested by centrifugation at 2000 *g* for 10 min at 4 °C.

The pharmacokinetic parameters were calculated based on individual concentration-time data using WinNonlin 8.1 software (Certara, Princeton, NJ, USA) under a non-compartmental model. The parameters included time to peak concentration (T_max_), peak concentration (C_max_), area under the concentration-time curve (AUC_INF_obs_, the area under the curve from the time of administration to the theoretical extrapolation of infinite time; AUC_last_, the area under the time curve from the beginning of the administration time to the last point), mean residence time (MRT_INF_obs_, the mean residence time extrapolated to infinity; MRT_last_, the mean residence time from the time of administration to the last concentration measured), volume of distribution (V_z_), clearance rate (Cl__obs_, clearance in vessel) and half-life (T_1/2_). Subsequently, the plasma pharmacokinetic characteristics of the drug in rats were analyzed, encompassing exposure levels, absorption characteristics, dose relationships, elimination rates, potential accumulation,.Comparisons between different groups were performed using the t-test, with a significance level set at P < 0.05.

### 2.7 Plasma protein binding

The plasma protein binding rate of (R)-ketamine hydrochloride was determined using the ultrafiltration method in mice, SD rats, Beagle dogs, cynomolgus monkeys, and humans. Warfarin was used as a positive control in this study. Test samples of (R)-ketamine hydrochloride were added to plasma from mice, SD rats, Beagle dogs, cynomolgus monkeys, and humans to prepare drug-containing plasma at concentrations of 0.1 µM, 1 μM, and 10 µM for each species. The drug-containing plasma from each species was incubated at 37 °C in a shaker for approximately 30 ± 1 min and then transferred to ultrafiltration tubes. After centrifugation, free and bound compounds were separated. The concentrations of free and total drugs were determined using the LC-MS/MS method. After incubation, 100 µL of drug-containing plasma was taken from the EP tube in triplicate (n = 3) to measure the total drug concentration (C_total_) in plasma.Additionally, 200 µL of drug-containing plasma was placed in ultrafiltration tube A (sample tube), while 200 µL of blank plasma was added to ultrafiltration tube B (control tube), each in triplicate (n = 3). The tubes were centrifuged at 10,000 g for 10 min at room temperature, and the filtrate and residue from tubes A and B were collected. The weight of the filtrate in tube A was measured, with 1 mg of filtrate converted to 1 µL for volume calculation. The residue from tube B was mixed with the filtrate from tube A and centrifuged at 700 *g* for 3 min at room temperature to obtain the free drug. The “measured signal” of sample A in the instrument, multiplied by the “dilution factor,” represented the free drug concentration (C_free_) in the drug-containing plasma.

The plasma incubation system was validated using warfarin as the positive control, with incubation and processing methods consistent with those of the test samples. The experimental results were considered valid if the binding rate of the positive control warfarin exceeded 90%.

$$Bound\left(\%\right)=\left(C_{total}-C_{free}\right)/C_{total}\times 100\%$$

### 2.8 Tissue distribution studies in rats

In this study, 24 SD rats were randomly divided into four groups, with six rats per group (male: female = 1:1). Prior to the experiment, the rats were not fasted and had free access to water. At each time point, the animals were administered a single intravenous injection of 7.5 mg/kg (R)-ketamine hydrochloride. At different time points post-administration (0.5 h, 2 h, 4 h, and 8 h), the rats were euthanized humanely euthanized by CO2 inhalation (30% flow rate of chamber volume per min). After blood was collected via cardiac puncture to prepare plasma. Approximately 0.6 mL of whole blood was collected to the tube containing EDTA-K2. The gathered blood samples were kept on ice until centrifugation. Plasma samples were harvested by centrifugation at 2000 *g* for 10 min at 4 °C. And then 13 tissues (heart, liver, spleen, lung, kidney, brain, stomach, duodenum, fat, muscle, ovary, uterus, and testis) were removed from the rats. These tissue samples were washed with saline and dried with filter paper. Aliquots of tissues were accurately weighed and then homogenized at a ratio of 1 g:10 mL (m/v) in 50% (v/v) methanol/water to obtain tissue homogenates. All biological samples were stored at −70 °C until analysis.

### 2.9 *In vitro* metabolic stability

The metabolic stability and metabolite profile of (R)-ketamine were evaluated using pooled microsomes from mice, rats, dogs, humans and monkeys. Phenacetin was employed as a positive control to monitor enzyme activity. A negative control was included in the assay, where phosphate buffer was added to the incubation system instead of NADPH, followed by mixing and incubation at 37 °C for 60 min, to investigate the potential presence of NADPH-independent metabolism.

The total incubation volume of 200 μL contained (R)-ketamine and phenacetin at a final concentration of 1 μM, consisting of 180 μL of drug-containing microsome working solution ((R)-ketamine at 1.11 µM, microsomal protein concentration of 0.556 mg/mL) or positive control working solution (phenacetin at 1.11 µM), along with 20 μL of NADPH working solution (10 mM), all diluted in phosphate buffered saline (PBS).The 180 μL of drug-containing microsome working solution or positive control working solution was pre-incubated at 37 °C for 5 min. The incubation was initiated by adding 20 µL of NADPH working solution followed by thorough mixing. For the drug-containing microsomes, the incubation was terminated at 0, 5, 10, 30, and 60 min by adding 400 μL of terminating solution (acetonitrile containing 10 ng/mL verapamil as internal standard). For the liver microsomes containing the positive control, the incubation was terminated at 10 and 60 min by adding 400 µL of terminating solution. A negative control was also included in the experiment. The incubation system was mixed with phosphate buffer instead of NADPH and incubated at 37 °C for 60 min. The resulting supernatant was mixed with deionized water at a 1:1 ratio by vortexing prior to determination of the peak area ratios of (R)-ketamine and phenacetin to verapamil.

A semi-quantitative approach was employed, where the peak area ratio of analyte to internal standard represented the remaining amount of the (R)-ketamine. The logarithmic value [Ln (R%)] of the remaining percentage of (R)-ketamine at time T relative to T0 was subjected to linear regression against reaction time T to calculate the slope.T_1/2_

$$T_{1/2}=0.693/Ke$$

$${CL}_{\mathrm{int}\;in\;vitro}=K/C_{microsomes\;}$$

$${CL}_{\mathrm{int}\;in\;vivo}={CL}_{\mathrm{int}\;in\;vitro}\times microsomes\;yield\times liver\;weight$$

The parameter C_microsomes_ represents the protein concentration (0.5 mg/mL) in the incubation system. A microsome yield (45 mg protein/g liver) of all species was used. The corresponding liver weights were approximately 87.5, 40, 32, 30, and 25.7 g/kg body weight for mice, rats, dogs, monkeys, and humans, respectively (Davies and Morris, 1993). Then, the scaled *in vivo* intrinsic clearance values were employed to estimate systemic clearance (equivalent to hepatic clearance) through application of the well-stirred venous equilibrium model.

$${CL}_{sys}={CL}_{\mathrm{int}\;in\;vitro}\times Q_{H}/\left(Q_{H}+{CL}_{\mathrm{int}\;in\;vivo}\right)$$

Q_H_ is the hepatic blood flow: 90 mL/min/kg for mice, 55.2 mL/min/kg for rats, 30.9 mL/min/kg for dogs, 43.6 mL/min/kg for monkeys, and 20.7 mL/min/kg for humans (Davies and Morris, 1993).

### 2.10 Metabolite profiling

After incubating (R)-ketamine with liver microsomes, the resulting samples were analyzed by LC-UV-MS^n^ (n = 1–2).MassLynx and MetaboLynx™ software was used to identify the metabolites of (R)-ketamine. A 10.0 µM working solution of (R)-ketamine hydrochloride was incubated with liver microsomes from mice, rats, dogs, monkeys, and humans at 37.0 °C for 30 min. Negative control and blank solvent groups were included. By comparing the primary mass spectrometry chromatograms of the incubated ones with the blank, the major differential components (potential metabolites) were identified. These potential metabolites were then subjected to secondary mass spectrometry analysis and structural identification to infer their structures.

The metabolites were detected and identified in liver microsomes from mice, rats, dogs, monkeys, and humans. The structures of the metabolites were elucidated by comparing the fragment ions of the parent compound and its metabolites based on their precise molecular weights and molecular formulas.The relative abundance of (R)-ketamine and its metabolites was calculated as the percentage of their respective mass spectral peak areas relative to the total mass spectral peak areas of all drug-related components.

### 2.11 Excretion studies in rats

A total of 22 SD rats were randomly divided into two groups. Group 1 consisted of 16 rats (male: female = 1:1) for bile excretion studies, while Group 2 included six rats for urine and feces excretion studies (male: female = 1:1). Both groups were administered a single intravenous injection of 7.5 mg/kg (R)-ketamine hydrochloride.

For Group 1, on day 1 after bile duct cannulation surgery, six male and six female SD rats with good postoperative recovery were selected for drug administration. Bile was collected at the following time points: pre-dose (0), 0–2 h, 2–4 h, 4–8 h, 8–24 h, 24–32 h, 32–48 h, 48–56 h, and 56–72 h post single dose. The bile samples were collected into centrifuge tubes and stored at −70 °C. For Group 2, urine and feces were collected at the following time: pre-dose (0), 0–2 h, 2–4 h, 4–8 h, 8–24 h, 24–32 h, 32–48 h, 48–56 h, and 56–72 h post single dose. Following urine sample collection during the 56–72 h period, the metabolic cage was rinsed three times with 20 mL of sterile deionized water. The used water was collected, measured for volume, aliquoted according to urine sample collection requirements, and stored with recorded volumes. Approximately 3 mL of urine was collected to the centrifuge tube. The gathered urine samples were kept in −70 °C. Fecal samples from each time interval were weighed after removing feed residues, and the entire quantity was collected in labeled storage tubes and then stored at −70 °C.

The concentrations of (R)-ketamine in bile, urine, and feces were quantified using a validated LC-MS/MS method, with a lower limit of quantification (LLOQ) of 1 ng/mL for (R)-ketamine. The total excretion amounts and excretion rates for each time interval were calculated.

### 2.12 Prediction of human pharmacokinetic parameters using allometric scaling

The allometric scaling (AS) approach was employed to predict human plasma total clearance (CL) based on preclinical data from different species (rats and dogs). The mean value of unbound plasma fraction (fu) was applied to transform CL to the corresponding free drug clearance (CLu). The following formula was applied, with the maximum lifespan potential (MLP) incorporated as a correction factor according to the rule of exponents (RoEs), to scale CLu from preclinical species to humans (Mahmood and Balian, 1996). Here, BW represents the body weight of standard species (0.2 kg for rats, 10 kg for dogs, and 70 kg for humans), a denotes the allometric coefficient, and b is the allometric exponent.

$${CL}_{u}=CL/f_{u}$$

$${CL}_{u}\times MLP=a\times {BW}^{b}$$

$$\log\left({CL}_{u}\times MLP\right)=\log\;a+b\times logBW$$

## 3 Results and discussion

### 3.1 Plasma pharmacokinetics

#### 3.1.1 (R)-ketamine plasma pharmacokinetics in rats

**FIGURE 1:**
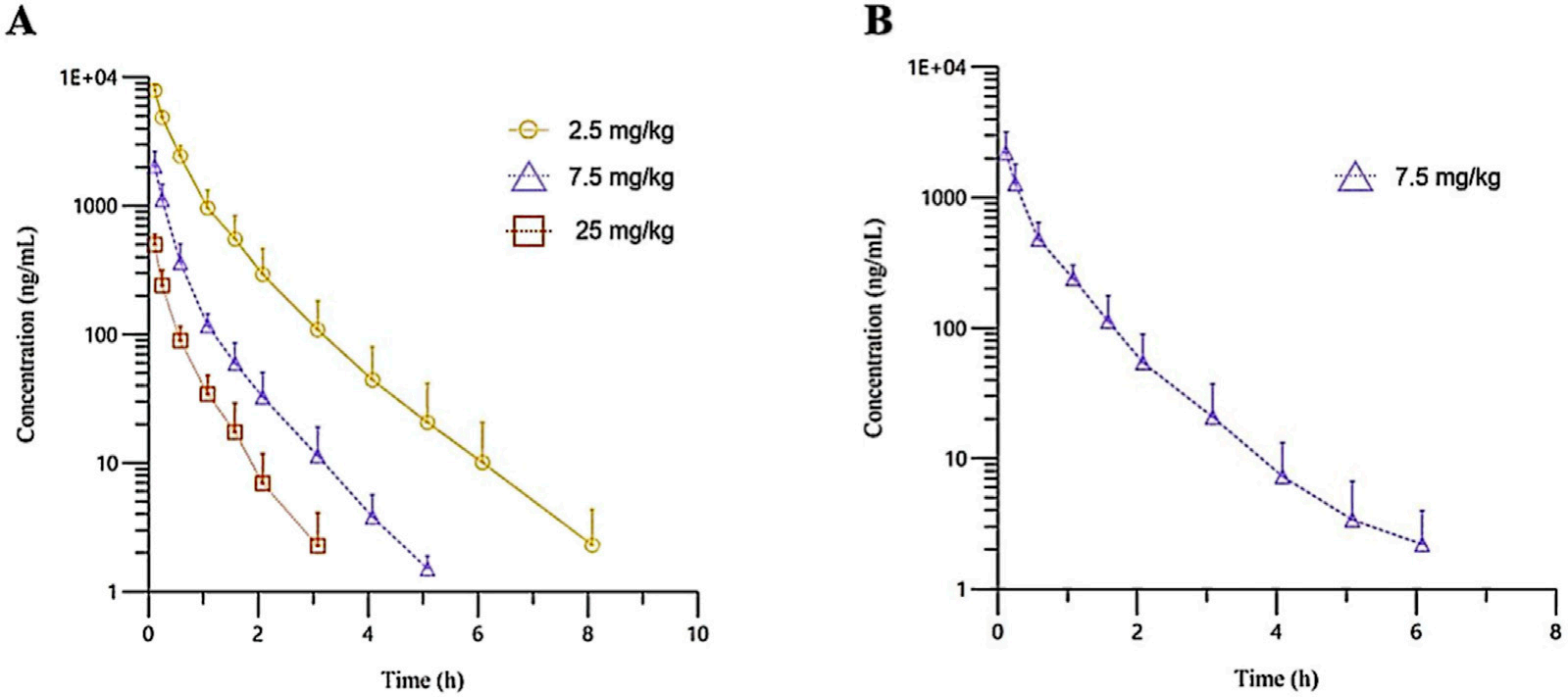
The mean plasma concentration-time profiles of (R)-ketamine in SD rats were plotted following a single intravenous administration of 2.5 mg/kg and 25 mg/kg (**(A)**, n = 6), as well as repeated administration of 7.5 mg/kg (R)-ketamine hydrochloride (**(B)**, n = 6). All data are expressed as the mean ± standard deviation. Statistical significance was determined using the t-test, with P < 0.05 considered statistically significant. All pre-dose blood sample concentrations were below the quantification limit (BQL).

The plasma pharmacokinetic (PK) parameters of (R)-ketamine were determined following single and repeated intravenous administration in both male and female rats. All pre-dose blood samples showed concentrations below the quantification limit. The PK parameters are summarized in Table 1 and the plasma concentration-time profiles are illustrated in Figure 1.Following single intravenous administration of (R)-ketamine hydrochloride injection at doses of 2.5, 7.5, and 25 mg/kg (1:3:10) to Sprague-Dawley rats, the Cmax and systemic exposure of (R)-ketamine were comparable between male and female rats. The increases of C_max_, AUC_last_, and AUC_INF_obs_ across dose groups exceeded the corresponding dose increments, demonstrating nonlinear pharmacokinetic characteristics.The dose-exposure relationships for (R)-ketamine pharmacokinetic parameters (C_max_, AUC_last_, and AUC_INF_obs_) were fitted using a power function model for low, medium, and high dose groups, yielding slope values (β) of 0.976, 0.986, and 0.986, respectively. The 90% confidence intervals for these slope values were [1.086, 1.320], [1.257, 1.454], and [1.255, 1.451], respectively. A proportional relationship between the increase in exposure and the increase in dose is concluded when the 90% confidence interval of β falls within the range of 80%–125%. After repeated intravenous administration of 7.5 mg/kg (R)-ketamine hydrochloride injection, the AUC_last_ and AUC_INF_obs_ of (R)-ketamine following the last dose were 1.29-fold those after the first administration, while C_max_ was 1.08-fold higher. These results indicate no accumulation of (R)-ketamine in SD rats following once-daily administration of 7.5 mg/kg (R)-ketamine hydrochloride injection for seven consecutive days. An independent samples t-test was conducted on the primary exposure parameters of (R)-ketamine within each group, including AUC, C_max_, Cl__obs_, and T_1/2_. The results indicated that in Group 3, male animals showed slightly higher AUC_INF_obs_ and AUC_last_ values compared to females (p < 0.05, with ratios of 0.725 and 0.726, respectively). No statistically significant differences (p > 0.05) were observed in other primary pharmacokinetic parameters between male and female animals in other group.The male/female ratios of major pharmacokinetic parameters (AUC, C_max_, Cl__obs_, T_1/2_) for (R)-ketamine in Groups 1–3 ranged from 0.725 to 1.39, indicating no significant gender-based differences in the primary pharmacokinetic parameters.

**TABLE 1:** The mean pharmacokinetic parameters of (R)-ketamine in plasma were summarized for SD rats following a single intravenous administration of 2.5 mg/kg and 25 mg/kg, as well as repeated administration of 7.5 mg/kg (R)-ketamine hydrochloride.

		2.5 mg/kg		7.5 mg/kg				25 mg/kg	
		Day 1		Day 1		Day 7		Day 1	
Parameter	Units	Male	Female	Male	Female	Male	Female	Male	Female
C_max_	ng/mL	527 ± 137	473 ± 57.0	2297 ± 266	1775 ± 831	2233 ± 428	2178 ± 1484	8060 ± 312	7713 ± 1080
T_max_	h	0.116 ± 0	0.116 ± 0	0.116 ± 0	0.116 ± 0	0.116 ± 0	0.116 ± 0	0.116 ± 0	0.116 ± 0
AUC_last_	h*ng/mL	195 ± 18.8	183 ± 41.0	817 ± 138	778 ± 293	923 ± 165	1131 ± 363	3617 ± 527	4987 ± 283
AUC_INF_obs_	h*ng/mL	196 ± 18.8	184 ± 41.4	818 ± 138	780 ± 293	924 ± 165	1112 ± 344	3620 ± 524	4993 ± 284
T_1/2_	h	0.522 ± 0.100	0.714 ± 0.0827	0.683 ± 0.126	0.656 ± 0.129	0.683 ± 0.0861	0.802 ± 0.110	1.16 ± 0.329	0.907 ± 0.0615
MRT_last_	h	0.366 ± 0.0528	0.540 ± 0.0953	0.371 ± 0.0363	0.604 ± 0.221	0.463 ± 0.0101	0.795 ± 0.455	0.524 ± 0.0927	0.795 ± 0.136
MRT_INF_obs_	h	0.383 ± 0.0479	0.576 ± 0.0936	0.381 ± 0.0339	0.616 ± 0.223	0.471 ± 0.0106	0.806 ± 0.462	0.530 ± 0.0912	0.802 ± 0.141
Vz__obs_	mL/kg	9717 ± 2310	10,667 ± 9143	9410 ± 3381	6793 ± 6336	8280 ± 2470	8650 ± 4635	11,860 ± 4312	6567 ± 661
Cl__obs_	mL/h/kg	12,800 ± 1277	10,700 ± 8577	9343 ± 1619	10,913 ± 5190	8300 ± 1591	7233 ± 2831	6993 ± 942	5020 ± 282

Data are expressed as mean ± SD (n = 3).

#### 3.1.2 (R)-ketamine plasma pharmacokinetics in beagle dogs

**FIGURE 2:**
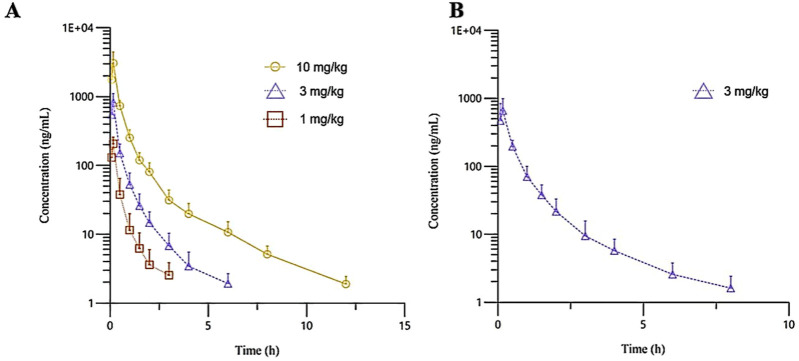
The mean plasma concentration-time curves of R-ketamine in Beagle dogs following a single intravenous administration of 1, 3, and 10 mg/kg (**(A)**, n = 6), as well as repeated administration of 3 mg/kg of (R)-ketamine hydrochloride injection (**(B)**, n = 6). All data are expressed as the mean ± standard deviation. Statistical significance was determined using the t-test, with P < 0.05 considered statistically significant. All pre-dose blood sample concentrations were below the quantification limit (BQL).

The plasma pharmacokinetic (PK) parameters of (R)-ketamine were determined following single intravenous injection and single as well as multiple oral administrations in both male and female Beagle dogs. All pre-dose blood samples showed concentrations below the quantification limit. The PK parameters are summarized in Table 2, and the plasma concentration-time profiles are illustrated in Figure 2. Following single intravenous administration of (R)-ketamine hydrochloride injection at doses of 1, 3, and 10 mg/kg (1:3:10) to Beagle dogs, the Cmax and systemic exposure of (R)-ketamine were comparable between male and female dogs. The increases of (R)-ketamine in C_max_, AUC_last_, and AUC_INF_obs_ across dose groups exceeded the corresponding dose increments, demonstrating nonlinear pharmacokinetic characteristics.The dose-exposure relationships for (R)-ketamine pharmacokinetic parameters (C_max_, AUC_last_, and AUC_INF_obs_) were fitted using a power function model for low, medium, and high dose groups, yielding slope values (β) of 0.572, 0.922, and 0.918, respectively. The 90% confidence intervals for these slope values were [0.388, 0.756], [0.813, 1.031], and [0.811, 1.024], respectively. A proportional relationship between the increase in exposure and the increase in dose is concluded when the 90% confidence interval of β falls within the range of 80%–125%. After repeated intravenous administration of 3 mg/kg (R)-ketamine hydrochloride injection, the AUC_last_ and AUC_INF_obs_ of (R)-ketamine following the last dose were 0.83-fold and 0.84-fold those after the first administration, respectively, while C_max_ was 0.67-fold higher. These results indicate no accumulation of (R)-ketamine in Beagle dogs following once-daily administration of 3 mg/kg (R)-ketamine hydrochloride injection for seven consecutive days. An independent samples t-test was conducted on the primary exposure parameters of (R)-ketamine within each group, including AUC, C_max_, Cl__obs_, and T_1/2_. The results demonstrated that in Group 3, male animals exhibited slightly higher C_max_ and AUC_last_ values compared to females (p < 0.05, with ratios of 2.14 and 1.71, respectively), while Cl__obs_ was slightly lower in males (p < 0.05, ratio 0.59). No statistically significant differences (p > 0.05) were observed in other primary pharmacokinetic parameters between male and female animals in other group.The male/female ratios of major pharmacokinetic parameters (AUC, C_max_, Cl__obs_, T_1/2_) for (R)-ketamine in Groups 1–3 ranged from 0.59 to 2.14. Gender differences in the pharmacokinetic parameter C_max_ were observed in Group 3, which may be attributed to metabolic saturation. The expression or activity of key enzymes responsible for metabolizing this drug may be lower in male Beagle dogs. At lower doses, the capacity of these enzymes is sufficient to process the drug, thus showing no significant differences. However, at higher doses, these enzymes in male dogs become saturated first. Gender-specific considerations regarding the therapeutic window and maximum tolerated dose should be incorporated into clinical study design.

**TABLE 2:** The mean pharmacokinetic parameters of (R)-ketamine in Beagle dogs following a single intravenous administration of 1 mg/kg, 3 mg/kg, and 10 mg/kg (R)-ketamine hydrochloride.

		1 mg/kg		3 mg/kg				10 mg/kg	
		Day 1		Day 1		Day 7		Day 1	
Parameter	Units	Male	Female	Male	Female	Male	Female	Male	Female
C_max_	ng/mL	203 ± 35.0	209 ± 72.6	935 ± 298	712 ± 258	674 ± 311	664 ± 447	4710 ± 904	1950 ± 435
T_max_	h	0.167 ± 0	0.167 ± 0	0.167 ± 0	0.167 ± 0	0.167 ± 0	0.139 ± 0.0483	0.167 ± 0	0.167 ± 0
AUC_last_	h*ng/mL	79.2 ± 11.2	85.8 ± 17.5	79.2 ± 11.2	85.8 ± 17.5	348 ± 93.8	353 ± 209	1810 ± 419	1061 ± 216
AUC_INF_obs_	h*ng/mL	81.0 ± 11.4	87.5 ± 18.6	81.0 ± 11.4	87.5 ± 18.6	351 ± 93.2	356 ± 210	1820 ± 419	1063 ± 212
T_1/2_	h	0.983 ± 0.250	0.745 ± 0.606	0.885 ± 0.184	1.24 ± 0.345	1.46 ± 0.258	1.67 ± 1.20	2.50 ± 0.304	2.11 ± 0.564
MRT_last_	h	0.335 ± 0.114	0.376 ± 0.349	0.373 ± 0.187	0.523 ± 0.181	0.603 ± 0.218	0.671 ± 0.277	0.623 ± 0.0168	0.768 ± 0.203
MRT_INF_obs_	h	0.431 ± 0.149	0.445 ± 0.420	0.410 ± 0.209	0.584 ± 0.196	0.676 ± 0.215	0.743 ± 0.327	0.684 ± 0.0149	0.843 ± 0.183
Vz__obs_	mL/kg	15,967 ± 6152	11,363 ± 6858	10,663 ± 3682	17,133 ± 2454	19,133 ± 7731	19,020 ± 8876	20,167 ± 2616	29,100 ± 7848
Cl__obs_	mL/h/kg	12,500 ± 1652	11,767 ± 2228	8220 ± 1115	10,243 ± 3568	9000 ± 2605	10,490 ± 5466	5677 ± 1166	9650 ± 2043

Data are expressed as mean ± SD (n = 3).

### 3.2 Distribution

#### 3.2.1 Plasma protein binding

The plasma protein binding rates of warfarin in mice, rats, dogs, monkeys, and humans were all greater than 97%, indicating that the test system was effective and met the acceptance criteria. The plasma protein binding rates of (R)-ketamine in mice, rats, dogs, monkeys, and humans are presented in Table 3. In summary, at the three tested concentrations (0.1 µM, 1 μM, and 10 µM), (R)-ketamine hydrochloride exhibited moderate binding in dog and human plasma. In mouse and rat plasma, it showed moderate binding at concentrations of 0.1 µM and 1 μM, but low binding at 10 µM. In monkey plasma, it exhibited moderate binding at 1 μM, but low binding at 0.1 µM and 10 µM. No significant correlation was observed between the plasma protein binding rate of (R)-ketamine hydrochloride and drug concentration across the tested species.

**TABLE 3:** The protein binding rate of (R)-ketamine hydrochloride in plasma across different species. (n = 3).

Species	Concentration (μM)	The plasma protein rates (%)
Mice	0.1	67.82 ± 9.12
	1	56.31 ± 4.23
	10	34.61 ± 6.26
Rats	0.1	56.38 ± 10.73
	1	56.52 ± 9.57
	10	32.59 ± 8.02
Dogs	0.1	60.90 ± 8.31
	1	60.26 ± 2.66
	10	54.17 ± 4.53
Monkeys	0.1	46.91 ± 8.64
	1	58.57 ± 4.84
	10	41.12 ± 7.22
Humans	0.1	56.71 ± 3.47
	1	70.07 ± 5.39
	10	57.90 ± 11.89

Data are expressed as mean ± SD (n = 3).

#### 3.2.2 Tissue distribution in rats

**FIGURE 3:**
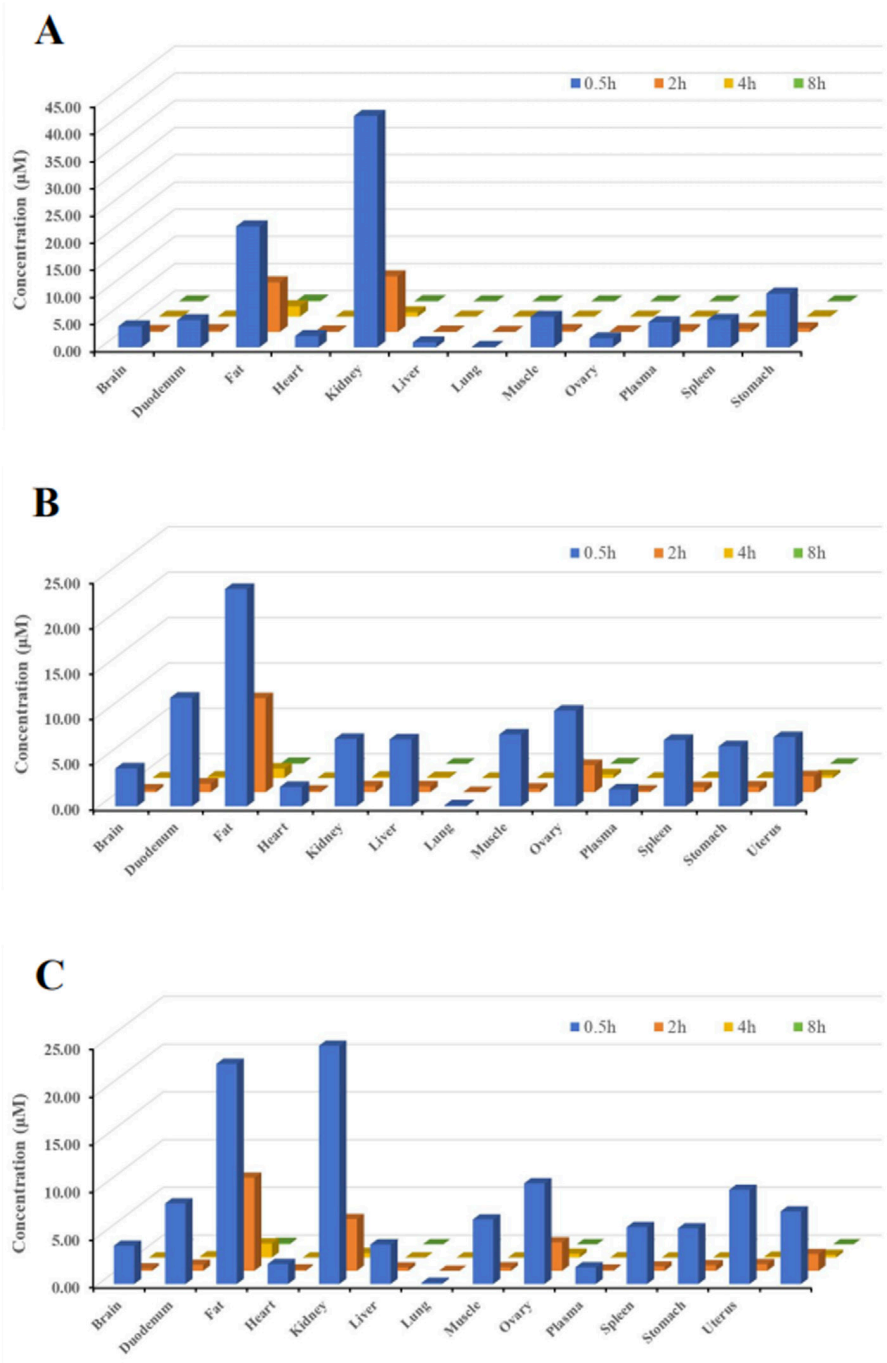
Tissue distribution of (R)-ketamine at 0.5 h, 2 h, 4 h, and 8 h after a single intravenous injection (7.5 mg/kg). (**(A)**, n = 6) The tissue concentration of (R)-ketamine in male rats. (**(B)**, n = 6) The tissue concentration of (R)-ketamine in female rats. ((**C**), n = 6) The average tissue concentration of (R)-ketamine in male and female rats. All data are expressed as the mean concentration.

The primary objective of this study was to investigate the tissue distribution characteristics of (R)-ketamine hydrochloride in SD rats following a single intravenous injection (7.5 mg/kg).As shown in Figure 3, (R)-ketamine was detectable in all examined tissues of both male and female rats, with exposure levels in most tissues exceeding those in plasma, indicating extensive tissue distribution. No significant sex-related differences in tissue exposure were observed.(R)-ketamine exhibited the highest accumulation in adipose tissue and kidney, with exposure approximately 24-fold and 18-folds higher than in plasma. The relative tissue-to-plasma exposure ratios in descending order were as follows:Ovary, testis, uterus, duodenum (five to nine times that of plasma) >Muscle, stomach, spleen, liver, brain, heart (two to four times that of plasma) >Heart (1.2 times that of plasma) >Plasma > Lung (9% that of plasma).(R)-ketamine reached peak concentrations (T_max_) in tissues at 0.5 h post-dose. By 4 h post-administration, tissue concentrations declined to <1/15 of peak levels, suggesting no significant long-term accumulation in either sex.Additionally, the brain-to-plasma AUC ratio ranged from 1.57 to 2.25, demonstrating effective blood-brain barrier (BBB) penetration of (R)-ketamine.

### 3.3 Metabolism

#### 3.3.1 *In vitro* metabolic stability in liver microsomes

Under NADPH-free conditions, the remaining percentages of (R)-ketamine after 60 min of incubation in liver microsomes from mice, rats, dogs, monkeys, and humans were 94.89%, 103.69%, 90.99%, 93.84% and 94.47%, respectively (Supplementary Table S1). The remaining percentages of (R)-ketamine all exceeded 90%, indicating the absence of NADPH-independent metabolism in the liver microsome incubation systems across these species.

Under NADPH-supplemented conditions, as shown in Table 4, the half-lives of phenacetin in liver microsomes from mice, rats, dogs, monkeys, and humans were 9.5 min, 60.9 min, 42.5 min, 41.6 min and 92.1 min, respectively. The calculated intrinsic clearance values in liver microsomes were 145.4 μL/min/mg, 22.8 μL/min/mg, 32.7 μL/min/mg, 33.3 μL/min/mg and 15.0 μL/min/mg for mice, rats, dogs, monkeys and humans, respectively. The calculated intrinsic clearance value of the positive control phenacetin are consistent with reference data reported in the literature (Shimada et al., 1997), confirming the stability and validity of the liver microsome incubation system. The *in vitro* half-lives of (R)-ketamine in liver microsomes from mice, rats, dogs, monkeys, and humans were 15.6 min, 4.8 min, 0.8 min, 3.0 min and 30.0 min, respectively. The corresponding *in vivo* clearance values were 71.6, 49.9, 30.5, 40.8 and 14.9 mL/min/kg, with hepatic extraction rates (ER) of 0.80, 0.90, 0.99, 0.94, and 0.72 for mice, rats, dogs, monkeys and humans, respectively. All hepatic extraction rate (ER) values exceeded 0.7. ER values of less than 0.3, greater than 0.7, and between 0.3 and 0.7 are classified as slow, fast and intermediate metabolism. These results demonstrate that (R)-ketamine hydrochloride undergoes rapid metabolism in liver microsomes across all tested species.

**TABLE 4:** The metabolic stability of (R)-ketamine hydrochloride in liver microsomes across various species in in NADPH-dependent incubation systems.

Compounds	Species	T_1/2_	CL_int_ _*in vitro*_	CL_int_ _*in vivo*_	CL_sys_	ER
		min	µL/min/mg	mL/min/kg	µL/min/mg	
(R)-ketamine	Mice	15.6	88.8	349.7	71.6	0.80
	Rats	4.8	286.7	516.1	49.9	0.90
	Dogs	0.8	1818.3	2618.3	30.5	0.99
	Monkeys	3.0	465.6	628.6	40.8	0.94
	Humans	30.0	46.1	53.4	14.9	0.72
Phenacetin	Mice	9.5	145.4	572.4	77.8	0.86
	Rats	60.9	22.8	41.0	23.5	0.43
	Dogs	42.5	32.7	47.0	18.6	0.60
	Monkeys	41.6	33.3	45.0	22.1	0.46
	Humans	92.1	15.0	17.4	9.5	0.46

CL_int, *in vitro*_, *in vitro* intrinsic clearance; CL_int, *in vivo*_, *in vivo* intrinsic clearance; CL_sys_, systemic clearance; ER, extraction ratio.

#### 3.3.2 Metabolite profiling

**FIGURE 4:**
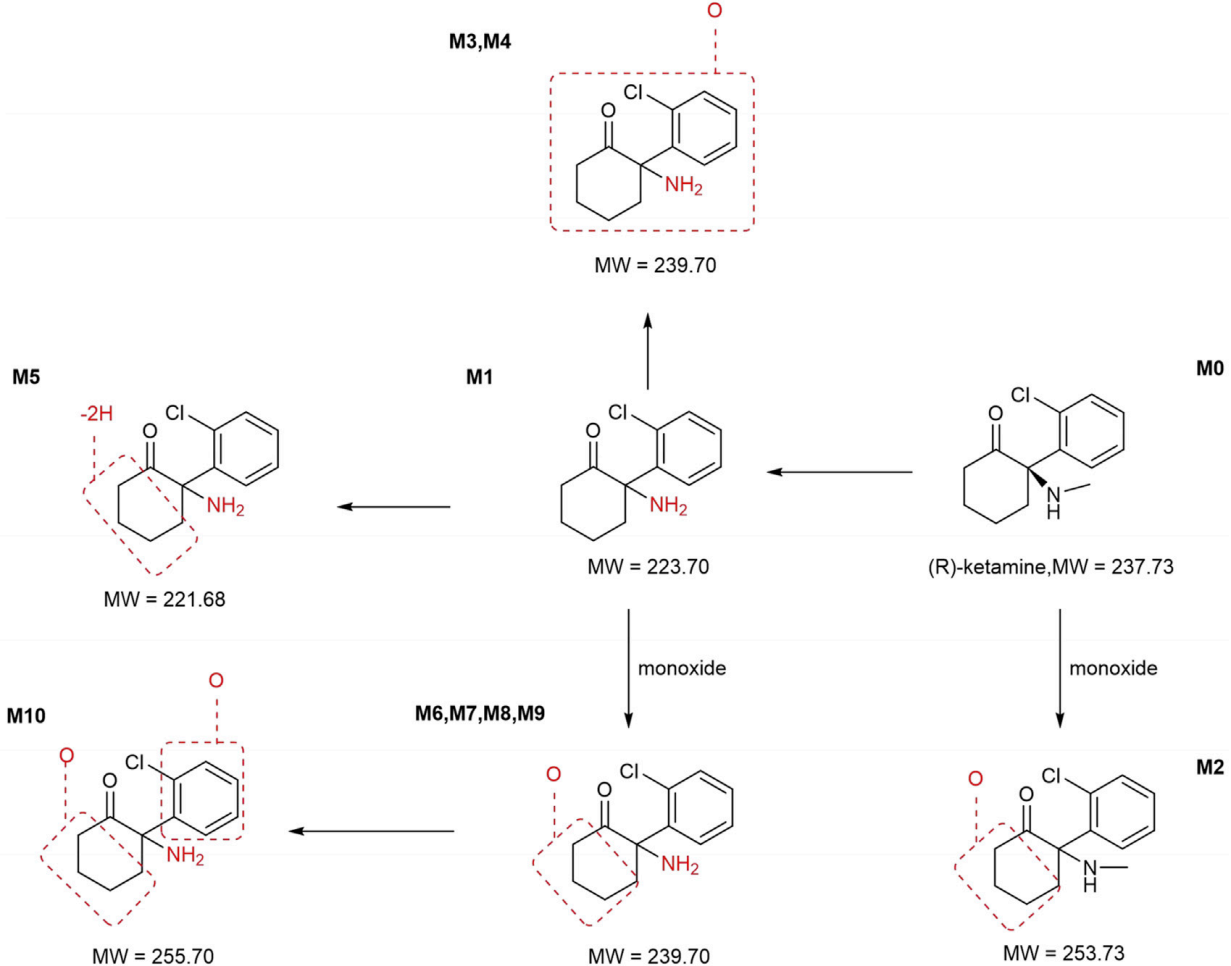
Metabolic pathway analysis of R-ketamine. MW, Molecular Weight.

In addition to the parent compound (R)-ketamine (Molecular Weight (MW) = 237.73), a total of 10 metabolites were detected and identified in liver microsomes from mice, rats, dogs, monkeys, and humans using LC-UV-MS^n^ (n = 1–2) technology. The metabolic pathways included oxidation, demethylation, dehydrogenation, and other transformations. Detailed information on each metabolite is provided in Table 5, and the metabolic pathway analysis is illustrated in Figure 4.

**TABLE 5:** Summary of the relative abundance and biotransformation pathways of (R)-ketamine and its metabolites.

Metabolites	Metabolic pathways	Proportion (%)				
		Mice	Rats	Dogs	Monkeys	Humans
M0	NA	79.7	+	+	2.55	68.9
M1	P-CH_2_	18.3	29.2	9.35	56.3	29.4
M2	P + O	+	0.985	+	0.051	+
M3	P-CH_2_+O	ND	2.21	ND	ND	ND
M4	P-CH_2_+O	0.570	0.442	1.30	0.082	0.202
M5	P-CH_2_-2H	ND	1.05	0.176	0.783	+
M6	P-CH_2_+O	+	4.31	1.27	2.18	0.0470
M7	P-CH_2_+O	1.18	29.5	66.5	14.2	0.564
M8	P-CH_2_+O	+	9.43	20.9	18.5	0.627
M9	P-CH_2_+O	0.177	20.5	0.331	5.39	0.257
M10	P-CH_2_+2O	0.106	2.51	0.102	ND	ND

P, (R)-ketamine; NA, inapplicable; +, Detected only by mass spectrometry; ND, not detected.

In mouse liver microsomes, eight metabolites (M6, M2, M4, and M6-M10) were detected. In rat liver microsomes, all 10 metabolites (M1-M10) were identified. In dog liver microsomes, nine metabolites (M1-M2 and M4-M9) were observed. In monkey liver microsomes, eight metabolites (M1 and M3-M9) were detected. In human liver microsomes, eight metabolites (M1 and M3-M9) were identified. Compared to other animal species, all metabolites detected in human liver microsomes were also found in the four tested animal species, indicating that no unique metabolites were formed in human liver microsomes after 30 min of incubation at 37 °C.

After incubation, the highest residual levels of the parent drug (M0) were observed in human (68.9%) and mouse (79.7%) incubation systems, whereas the M0 was extensively metabolized in monkeys (2.55%), dogs and rats (trace amounts). These findings suggest potential species differences in the metabolic rate of (R)-ketamine. This indicates that (R)-ketamine undergoes slower metabolism and exhibits a longer half-life in humans, suggesting its potential suitability for extended dosing intervals. The liver microsome metabolic profiles revealed that (R)-ketamine undergoes metabolism via N-demethylation to form (R)-norketamine (M1) and mono-oxidation/hydroxylation. The N-demethylation pathway generating M1 showed the highest relative abundance in monkeys (56.3%) and humans (29.4%), followed by rats (29.2%), while lower levels were observed in dogs (9.35%) and mice (18.3%). The high abundance of M1 in humans suggests its potential role in efficacy and toxicity. Clinical studies should focus on the dynamic changes of both the parent drug and the active metabolite M1. Based on existing studies, (R)-ketamine is metabolized in the liver via cytochrome P450 enzymes. The activity and expression levels of CYP enzymes for N-demethylation (e.g., CYP3A4 and CYP2B6) vary among individuals. In clinical practice, particularly when co-administered with other drugs, therapeutic drug monitoring may be necessary to achieve personalized dosing.

### 3.4 Excretion in rats

**FIGURE 5:**
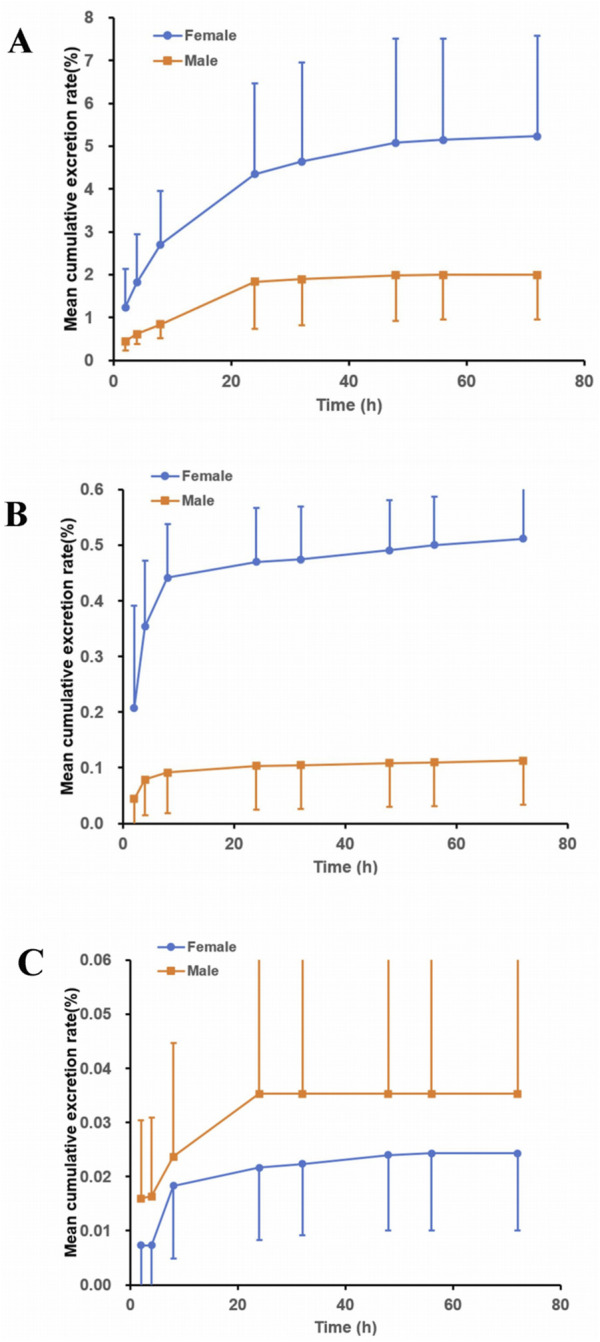
The mean cumulative excretion rate (%) of R-ketamine in bile (**(A)**, n = 6), urine (**(B)**, n = 6), and feces (**(C)**, n = 6) following a single intravenous administration of 7.5 mg/kg (R)-ketamine hydrochloride injection in SD rats. All data are expressed as the mean ± standard deviation.

The excretion pathways of (R)-ketamine were investigated following a single intravenous administration in intact and bile duct-cannulated rats. The mean cumulative excretion fraction curves are presented in Figure 5.The results demonstrated that after a single intravenous administration of 7.5 mg/kg (R)-ketamine hydrochloride to SD rats, the cumulative excretion rates of (R)-ketamine in bile, urine, and feces within 72 h were 3.613% ± 2.193%, 0.327% ± 0.221%, and 0.030% ± 0.018%, respectively. Following intravenous administration of 7.5 mg/kg (R)-ketamine hydrochloride to SD rats, (R)-ketamine was primarily excreted via bile, with cumulative excretion rates of 3.613% ± 2.193% within 72 h. This indicates that excretion of the parent compound via feces or urine is not the primary elimination pathway. Instead, the main elimination pathway is likely metabolic transformation, with subsequent excretion of metabolites.

### 3.5 Human PK prediction

After incorporating the unbound plasma fraction values and maximum lifespan potential (MLP) across different species, the allometric coefficient (a = 3981) and allometric exponent (b = 0.39) were derived from the clearance data of rats and Beagle dogs. The predicted human plasma clearance was 29.93 mL/min/kg, which was close to the reported human plasma clearance of 20.00 mL/min/kg (Persson, J. et al., 2002) (within two-fold error).

## 4 Conclusion

The pharmacokinetic characteristics of the (R)-ketamine injection have been thoroughly investigated, and these findings can provide valuable information for predicting the first-in-human dose and designing Phase I clinical trials. This study established an LC-MS/MS method for the quantitative detection of (R)-ketamine concentrations in various biological matrices and systematically evaluated the *in vivo* pharmacokinetic characteristics, distribution, metabolism, and excretion properties of (R)-ketamine. Based on preclinical PK data, the clearance of (R)-ketamine in humans was estimated. Furthermore, the plasma exposure of (R)-ketamine demonstrates nonlinear pharmacokinetic characteristics. A more conservative dose-escalation approach in clinical studies, such as using smaller incremental multiples or including more subjects in each dose group, is recommended to fully evaluate the PK behavior. Liver microsome metabolic stability studies indicated that (R)-ketamine is readily metabolized and eliminated in mouse, rat, dog, human, and monkey liver microsomes. (R)-ketamine demonstrated widespread tissue distribution in rats, with higher drug exposure in the kidneys compared to other organs, and its primary excretion pathway was through bile. In clinical trials, enhanced monitoring of liver and kidney function is necessary, with particular attention to the risks in patients with impaired liver or kidney function. This study provides comprehensive preclinical data for (R)-ketamine, supporting the prediction of the first-in-human dose and the design of Phase I clinical trials.

## Data Availability

The original contributions presented in the study are included in the article/Supplementary Material, further inquiries can be directed to the corresponding authors.
