# Supplementary material for: Preclinical pharmacokinetic characterization of (R)-ketamine injection, a novel antidepressant glutamatergic agent
Source: Front Pharmacol. 2025 Nov 21;16:1699304. doi: 10.3389/fphar.2025.1699304 (PMC12678371; doi:10.3389/fphar.2025.1699304)
Supplement: Supplementary file 1 [file DataSheet1.pdf]

Supplementary Material

1.1 Declaration of the ethical issue

Jiangsu Nhwa Pharmaceutical Co., Ltd. commissioned InnoStar Bio-tech Nantong Co., Ltd. to conduct preclinical animal studies involving animal ethics in this research, the signature page of the contract is provided in supplementary 1. The specific test numbers can be found in the official statement in supplementary 2 and 3. According to the contract agreement between the two parties, all intellectual property rights arising from the commissioned research belong to Jiangsu Nhwa Pharmaceutical Co., Ltd. Therefore, although the ethical approval for this study was issued by Innostar's IACUC, no researchers from Innostar were included in the author list.

合同编号：2021HMAP026

委托人甲方	名称(或姓名)	江苏恩华药业股份有限公司 (签章)			技术合同专用章<br>或<br>单位公章<br>2021.06.17
	法定代表人	孙彭生 (签章)			
	委托代理人	/ (签章)			
	联系(经办)人	孙庆弟 孙庆弟 (签章)			
	住所<br>(通讯地址)	徐州经济开发区杨山路18号	邮政编码		
	电话	0516-87661013			
	开户银行	徐州交行营业部			
	帐号	323600660010210095291			
研究开发人乙方	名称(或姓名)	益诺思生物技术南通有限公司(签章)			技术合同专用章<br>或<br>单位公章
	法定代表人	常艳 (签章)			
	委托代理人	/ (签章)			
	联系(经办)人	吴伟娜 (签章)			
	住所<br>(通讯地址)	江苏省海门区临江镇洞庭湖路100号	邮政编码	226133	
	电话	0513-82625622			
	开户银行	江苏银行股份有限公司海门支行			
	帐号	50320188000186621			

Supplementary 1. The signature page of the contract between Jiangsu Nhwa Pharmaceutical Co., Ltd. and InnoStar Bio-tech Nantong Co., Ltd. for conducting animal studies.

声明函

致江苏恩华药业股份有限公司：

鉴于江苏恩华药业股份有限公司（以下简称“委托方”）于益诺思生物技术南通有限公司（以下简称“受托方”）于 2021 年 6 月签署了关于“盐酸（R)氯胺酮临床前毒理学和药代动力学研究”项目的《技术开发合同》（合同编号：2021HMAP026），以下简称“合同”。根据委托方要求，对合同项下实际开展试验和知识产权条款，受托方特此声明如下：

1、根据合同约定和实际履约情况，委托方委托受托方进行过以下涉及动物伦理的临床前动物试验研究，具体试验编号、试验内容以及 IACUC 批准号具体信息如下：

（1）试验编号：H21025PK3，试验内容：SD 大鼠静脉注射给予盐酸（R）氯胺酮单次及重复给药药代动力学试验，IACUC 批准号为 H21025PK3：2021-714a。

（2）试验编号：H21025PK4，试验内容：Beagle 犬静脉注射给予盐酸（R）氯胺酮单次及重复给药药代动力学试验，IACUC 批准号为 H21025PK4：2021-711。

（3）试验编号：H21025TD1，试验内容：SD 大鼠静脉注射盐酸（R）氯胺酮注射液单次给药组织分布试验，IACUC 批准号为 H21025TD1：2021-715a。

（4）试验编号：H21025EX1，试验内容：SD 大鼠静脉注射盐酸（R）氯胺酮注射液单次给药排泄试验，IACUC 批准号为 H21025EX1：2021-712。

2、根据合同约定，因合同委托试验所产生的知识产权归委托方所有。

特此声明！

声明方：益诺思生物技术南通有限公司

日期：2025 年 7 月 31 日

Supplementary 2. The signature page of the contract between Jiangsu Nhwa Pharmaceutical Co., Ltd. and InnoStar Bio-tech Nantong Co., Ltd. for conducting animal studies - Chinese version.

Client: Jiangsu Nhwa Pharmaceutical Co., Ltd.
Contractor: Innostar Bio-tech Nantong Co., Ltd.
Declaration:
Jiangsu Nhwa Pharmaceutical Co., Ltd. (hereinafter referred to as the "Client") hereby engages Innostar Bio-tech Nantong Co., Ltd. (hereinafter referred to as the "Contractor") to conduct the following preclinical animal studies involving animal ethics. The specific study codes and descriptions are as follows:
Study Code: H21025PK3
Study Description: Pharmacokinetic study of single and repeated intravenous administration of (R)-ketamine hydrochloride in SD rats.
Study Code: H21025PK4
Study Description: Pharmacokinetic study of single and repeated intravenous administration of (R)-ketamine hydrochloride in Beagle dogs.
Study Code: H21025TD1
Study Description: Tissue distribution study of single intravenous administration of (R)-ketamine hydrochloride injection in SD rats.
Study Code: H21025EX1
Study Description: Excretion study of single intravenous administration of (R)-ketamine hydrochloride injection in SD rats.
The Institutional Animal Care and Use Committee (IACUC) overseeing the aforementioned studies is affiliated with Innostar Bio-tech Nantong Co., Ltd. The corresponding IACUC approval numbers are as follows:
H21025PK3: 2021-714a
H21025PK4: 2021-711
H21025TD1: 2021-715a
H21025EX1: 2021-712
Pursuant to the contractual agreement between both parties, all intellectual property rights arising from these commissioned works shall vest exclusively in Jiangsu Nhwa Pharmaceutical Co., Ltd.
This serves as an official declaration.

Supplementary 3. The signature page of the contract between Jiangsu Nhwa Pharmaceutical Co., Ltd. and InnoStar Bio-tech Nantong Co., Ltd. for conducting animal studies - English version.

1.2 Method validation for quantification of (R)-ketamine in SD rats and Beagle dogs plasma

1.2.1 Accuracy and precision

Four quality control (QC) samples at different concentrations contained LLOQ QC (1.00 ng/mL), LQC (3.00 ng/mL), MQC (75.0 ng/mL) and HQC (1500 ng/mL) were used to evaluate intra and inter-accuracy and precision from three independent analytical runs. The acceptance criteria required that both intra and inter-accuracy deviations at each concentration level should fall within ±15.0% (± 20.0% for LLOQ), while intra and inter-run precision should not exceed 15.0% (20.0% for LLOQ).

For (R)-ketamine in SD rats plasma, the intra-accuracy bias for all QC concentrations (excluding LLOQ QC) ranged from -6.3% to 8.6%, with intra-precision ranging from 0.9% to 11.1%. The inter-accuracy bias ranged from -4.6% to 2.6%, with inter-precision ranging from 4.1% to 8.8%. For the LLOQ QC of (R)-ketamine, the intra-accuracy bias ranged from 3.4% to 16.0%, with intra-precision

ranging from 10.4% to 13.1%. The inter-run accuracy bias was 5.1%, with an inter-run precision of 11.1%. For (R)-ketamine in Beagle dogs plasma, the intra accuracy bias for all QC concentrations (excluding LLOQ QC) ranged from -4.2% to 6.6%, with intra-precision ranging from 1.6% to 7.2%. The inter-accuracy bias ranged from -3.4% to 3.5%, with inter-precision ranging from 2.4% to 6.3%. For the LLOQ QC of (R)-ketamine, the intra-accuracy bias ranged from -11.7% to 6.8%, with intra-precision ranging from 5.9% to 11.0%. The inter-run accuracy bias was -0.6%, with an inter-run precision of 11.2%. The results demonstrate that the method exhibits satisfactory precision and accuracy.

1.2.2 Calibration curve and linearity

A linear regression model with a weighting factor of $1/x^2$ was used to establish the calibration curve, with quantification based on the linear relationship between theoretical concentrations and corresponding responses (peak area ratio of analytes to IS). The accuracy of calibration standards (CSs) should be within ±15%, except for the LLOQ which allows ±20%. In the final regression, at least six calibration levels and 75% of all CSs must meet the acceptance criteria.

(R)-ketamine demonstrated good linearity over the range of 1.00-2000 ng/mL in SD rats and Beagle dogs plasma. The correlation coefficients (R^2) were ≥0.9918 and ≥0.9940 for (R)-ketamine in SD rats and Beagle dogs plasma, respectively. The lower limits of quantification (1.00 ng/mL for (R)-ketamine) demonstrated adequate sensitivity during method validation.

1.2.3.Selectivity and Carryover

Selectivity was examined using six distinct lots of blank matrix to assess potential interference from endogenous components. The mean response of any interfering peak for the analyte must be ≤20.0% of the mean LLOQ response, while interfering peaks for the internal standard (IS) must be ≤5.0% of the mean IS response at LLOQ. Carryover was assessed by analyzing blank samples after injection of an upper limit of quantification (ULOQ) standard. The mean peak area in blank samples for either analyte or IS must not exceed 20.0% (analyte) or 5.0% (IS) of the corresponding mean response in LLOQ samples.

Interference from the internal standard to the analyte was within acceptable limits in SD rats and beagle dogs plasma. Carryover (%) was investigated by analyzing extracted blank samples after the ULOQ. No obvious carryover was observed in the blank plasma injected after the ULOQ sample. (2000 ng/mL for (R)-ketamine).

1.2.4.Matrix Effects

Matrix effects were evaluated by analyzing LQC (3.00 ng/mL) and HQC (1500 ng/mL) samples prepared in at least six individual blank matrix sources, with a minimum of three replicates per concentration level. The coefficient of variation (CV%) for the internal standard-normalized matrix factor at each concentration level should not exceed 15.0%.

At the low quality control concentration (LQC, 3.00 ng/mL) of (R)-ketamine in SD rats and Beagle dogs plasma, the mean IS-normalized matrix factor was 0.98 ± 0.02 with a relative standard deviation (RSD) of 2.0% and 1.00 ± 0.04 with RSD of 4.0%. At the high quality control concentration (HQC, 1500 ng/mL) in SD rats and Beagle dogs plasma , the mean IS-normalized matrix factor was 0.99 ± 0.02 with an RSD of 2.0% and 1.01 ± 0.04 with an RSD of 4.0%, respectively. The results meet the requirements for matrix effect evaluation.

1.2.5.Recovery

Recovery was assessed using matrix samples at low (LQC, 3.00 ng/mL), medium (MQC, 75.0 ng/mL), and high (HQC, 1500 ng/mL) concentration levels. The acceptance criterion required that the coefficient of variation (CV%) of recovery for the analyte at each concentration level should not exceed 15.0%.

The extraction recoveries of (R)-ketamine in SD rats and Beagle dogs plasma samples at low (3.00 ng/mL), medium (75.0 ng/mL), and high (1500 ng/mL) concentration levels were 99.4%-121.0% and 102.8%-107.7%, with CV values not exceeding 7.6%. The recovery of the internal standard tolbutamide in SD rats and Beagle dogs plasma were 96.7% and 98.6%, with CV values not exceeding 3.1%.

1.2.6.Stability

The stability of low and high concentration quality control samples (LQC and HQC) was evaluated under various storage conditions (room temperature and -70°C) for different time intervals, with a minimum of six replicates per concentration level. The acceptable value of QC samples should be within ±10.0% of the nominal values, with CV ≤15.0%.

The stability of (R)-ketamine in SD rats plasma was evaluated at both LQC (3.00 ng/mL) and HQC (1500 ng/mL) concentration levels.The analyte demonstrated stability in SD rats and Beagle dogs plasma contains ethylenediaminetetraacetic acid dipotassium salt (EDTA-K2) at least 22 and 23 hours at room temperature, respectively. Stability was maintained for at least 57 days for SD rats samples and 58 days for Beagle dogs samples when stored at -70°C. Both species samples met stability requirements after undergoing five freeze-thaw cycles (-70°C to room temperature).

1.3 In vitro metabolic stability in liver microsomes

Table S1 The metabolic stability of (R)-ketamine hydrochloride in liver microsomes across various species in NADPH-free incubation systems.

	Species	Time (min)	Peak area ratio ((R)-ketamine/verapamil)	R (%)
(R)-ketamine	Mice	0	20.271 ± 0.26	94.89
		60	19.235 ± 1.19	
	Rats	0	16.982 ± 0.26	103.69
		60	17.609 ± 2.67	
	Dogs	0	19.392 ± 2.06	90.99
		60	17.645 ± 1.18	
	Monkeys	0	19.000 ± 0.12	93.84
		60	17.830 ± 1.46	
	Humans	0	18.867 ± 0.71	94.47
		60	17.825 ± 0.57	

R, remaining percentage; Peak area ratio data are expressed as mean ± SD (n = 3).
